# Review: Insights on Current FDA-Approved Monoclonal Antibodies Against Ebola Virus Infection

**DOI:** 10.3389/fimmu.2021.721328

**Published:** 2021-08-30

**Authors:** Olivier Tshiani Mbaya, Philippe Mukumbayi, Sabue Mulangu

**Affiliations:** ^1^Clinical Monitoring Research Program Directorate, Leidos Biomedical Research, Frederick, MD, United States; ^2^Department of Molecular and Developmental Medicine, University of Siena, Siena, Italy; ^3^Global Medical Affairs, Ridgeback Biotherapeutics, Miami, FL, United States

**Keywords:** Ebola virus, antibodies, monoclonal, therapeutics, filovirus

## Abstract

The unprecedented 2013-2016 West Africa Ebola outbreak accelerated several medical countermeasures (MCMs) against Ebola virus disease (EVD). Several investigational products (IPs) were used throughout the outbreak but were not conclusive for efficacy results. Only the Randomized Controlled Trial (RCT) on ZMapp was promising but inconclusive. More recently, during the second-largest Ebola outbreak in North Kivu and Ituri provinces, Democratic Republic of the Congo (DRC), four IPs, including one small molecule (Remdesivir), two monoclonal antibody (mAb) cocktails (ZMapp and REGN-EB3) and a single mAb (mAb114), were evaluated in an RCT, the Pamoja Tulinde Maisha (PALM) study. Two products (REGN-EB3 and mAb114) demonstrated efficacy as compared to the control arm, ZMapp. There were remarkably few side effects recorded in the trial. The FDA approved both medications in this scientifically sound study, marking a watershed moment in the field of EVD therapy. These products can be produced relatively inexpensively and can be stockpiled. The administration of mAbs in EVD patients appears to be safe and effective, while several critical knowledge gaps remain; the impact of early administration of Ebola-specific mAbs on developing a robust immune response for future Ebola virus exposure is unknown. The viral mutation escape, leading to resistance, presents a potential limitation for single mAb therapy; further improvements need to be explored. Understanding the contribution of Fc-mediated antibody functions such as antibody-dependent cellular cytotoxicity (ADCC) of those approved mAbs is still critical. The potential merit of combination therapy and post-exposure prophylaxis (PEP) need to be demonstrated. Furthermore, the PALM trial has accounted for 30% of mortality despite the administration of specific treatments. The putative role of EBOV soluble Glycoprotein (sGP) as a decoy to the immune system, the virus persistence, and relapses might be investigated for treatment failure. The development of pan-filovirus or pan-species mAbs remains essential for protection. The interaction between FDA-approved mAbs and vaccines remains unclear and needs to be investigated. In this review, we summarize the efficacy and safety results of the PALM study and review current research questions for the further development of mAbs in pre-exposure or emergency post-exposure use.

## Introduction

The *Filoviridae* family includes two genera: *Marburgvirus* and *Ebolavirus*. These are enveloped viruses with a non-segmented, single-stranded, negative-sense RNA genome. The *Ebolavirus* genus has six virus species: Ebola virus (EBOV), *Sudan ebolavirus* (SUDV), *Taϊ Forest ebolavirus* (TAFV), *Reston ebolavirus* (RESTV), *Bundibugyo ebolavirus* (BDBV), and the recently described *Bombali ebolavirus* (BOMV). Both EBOV and SUDV were first described in 1976 in separate outbreaks in the DRC and Sudan, respectively (1–3), and are responsible for the greatest number of outbreaks. Since its first appearance, the majority of EVD epidemics have primarily occurred in Central Africa (4, 5). There has been no specific EVD treatment or cure for about 44 years (6). In early 2013, efforts started to identify MCMs to treat accidental laboratory exposure. The consensus to focus effort on mAbs, as a potential promising therapeutic, has been reached (7). Historical use of polyclonal antibodies to treat filovirus infection has shown some promising success. Convalescent sera were administered to patients with active EVD during the 1995 Kikwit, Zaire outbreak. The mortality reported out of the eight treated patients was 12.5%, a major reduction over the global mortality of EVD cases without specific medical intervention (8).The deadly Ebola outbreak in West Africa from 2013 to 2016 has spurred the development of many EVD MCMs. World Health Organization (WHO) convened in August 2014 to consider the use of unregistered interventions during the EBOV outbreak under expanded access protocol (EAP) (9). IPs have been identified based on extensive preclinical testing in animal models demonstrating post-exposure efficacy and on tracked record safety data from previous human studies. During this 2013-2016 West-Africa Ebola outbreak, several identified IPs have been used in non-control studies with a limited conclusion on efficacy. An RCT with ZMapp, a cocktail of mAbs, as the intervention arm was initiated late during the outbreak, and the results did not reach the pre-specified statistical threshold for efficacy against Ebola (10). Most recently, the second-largest EVD outbreak in history (3,470 cases; 2,287 deaths) occurred in the provinces of North-Kivu and Ituri/DRC. This outbreak began on August 1, 2018, following the DRC Ministry of Health’s official declaration, and ended on June 25, 2020, with an active transmission period of up to two years (4, 11–13). The difficulty in implementing and deploying public health measures was exacerbated by the political instability of the region and the high level of community mistrust of international and even national response teams (11, 13, 14). Additionally, the proximity of the North-Kivu and Ituri provinces to the Uganda borders created the potential for virus spreading to neighboring countries, as was seen during the 2013 West African EVD experience (4). Fortunately, this did not occur. Although critical challenges were encountered during this outbreak, joint effort of multidisciplinary teams, the traditional measures of prevention, and the innovative strategies, including IPs, were able to control the epidemic (11, 13, 14). The PALM RCT took place during this outbreak and evaluated the efficacy of 4 promising therapies against EBOV. Two IPs, mAb114 and REGN-EB3, successfully demonstrated efficacy against EBOV by significantly reducing the mortality rate of EVD compared to ZMapp (15). Although the efficacy results of mAb114 and REGN-EB3 were noticeable, 35.1% (61/174) and 33.5% (52/155) of the participants who received respectively mAb114 and REGN-EB3 died. The mortality was even higher, around 69.9% (51/73) and 63.6% (42/66) for mAb114 and REGN-EB3 respectively in the subset of participants presenting with high viral load (Ct ≤22) at baseline. Causes explaining this residual mortality may be related to the virus, the host, and even the intervention itself. Considering the future expanded use of those EVD FDA-approved therapies, continuous research efforts should be made to improve those products. In this review, we summarize the efficacy and safety results of the PALM study for the current FDA-approved mAbs therapeutics against EBOV and review ongoing research questions in further development of mAbs for improving efficacy in treatment or post-exposure prophylactic use.

## PALM Ebola RCT: Efficacy Results, Safety, and Room for Survival Improvement

Early during the North-Kivu and Ituri EVD epidemic, four IPs including a polymerase inhibitor (Remdesivir), two cocktails of mAbs (REGN-EB3 and ZMapp), and a single human mAb (mAb114) were rolled out under the Monitored Emergency Use of Unregistered Interventions (MEURI) protocol (16). While this protocol was not controlled, an RCT (clincaltrials.gov identifier NCT03729586), the PALM study, evaluating the efficacy and safety of those four MCMs, was initiated in November of 2018 (15). The target sample size was 725 patients (15). The primary endpoint was mortality at day 28. During the interim review of the Data Safety and Monitoring Board (DSMB) in August 2019, two products were identified to have crossed the pre-specified statistical threshold for efficacy against Ebola, at which point the DSMB recommended halting enrollment. Six hundred seventy-three patients out of a total of 681 enrolled patients received specific treatments. The global case fatality rate (CFR) reported during this outbreak was ~67%. The study results related to the primary outcome were mortality at 50% and 53% for ZMapp and Remdesivir, respectively after 28 days of follow up. Adult and pediatric patients receiving REGN-EB3 and mAb114 had 28-day CFRs of 34% and 35%, respectively. In the group of patients with high viral load (Ct ≤ 22.0), the CFR was 85% for ZMapp and Remdesivir, 64% and 70%, respectively for REGN-EB3 and mAb114. The overall survival improved in all therapeutic arms when the viral load was low (Ct>22) with a CFR of 25%, 29%, 10%, and 11%, respectively for ZMapp, Remdesivir, REGN-EB3, and mAb114 (15). Reported severe adverse events (SAE) possibly related to the trial drugs all resulted in death during the study. Four SAE in three patients were represented by gastrointestinal syndrome worsening, peri-infusional hypotension and hypoxia under ZMapp, and hypotension under Remdesivir. Following the DSMB review and recommendations, the study continued to enroll under two arms only (mAb114 and REGN-EB3) (15), which have demonstrated efficacy against EBOV and were recently approved by the US FDA for use in adults and pediatric EVD patients (17, 18). Importantly, improved survival during the PALM trial was noticed even in patients at high risk of disease. Also, the early arrival of EVD patients to the Ebola treatment center (ETC) was a determinant for survival when compared to patients admitted later in the disease course with risk of vital organ failure. Despite the PALM study finding these therapeutics to be highly effective against EBOV, some treated patients still succumbed with high viral load. Aggressive standard of care may help support vital functions and increase survival, as demonstrated by the 2013-2016 West African EVD cases transferred in specialized intensive unit care in Western countries.

Furthermore, based on the observation of seroconverted contacts who arrived late at the ETC, a field approach proposes using current FDA-approved therapeutics for post-exposure prophylaxis (PEP) on EVD contacts with a high risk of exposure. This approach is currently restricted to health care workers (HCW) with documented exposure risk (needle stick injury), and guidelines have been established for their prompt medical care management. Extending the use of current FDA-approved therapeutics for PEP at the community level may present an attractive option based on their easy parenteral administration and good tolerance. However, before being implemented on a large scale, the endeavor of PEP using specific mAbs in exposure to EBOV must be evaluated in a controlled study for proof of efficacy. A preliminary step in this concept will be to clearly define the “high-risk” contact in the health care facility and the community, knowing that less than 10% of contacts eventually seroconvert or develop the disease.

The persistence of EBOV has been previously reported in survivors with evidence of viral replication (19) and consecutive occurrence of sequelae. The molecular weight of mAbs limits their penetration into immune sanctuaries where EBOV persists. Combination therapy of EVD FDA-approved mAbs with promising antiviral small molecules may help to clear the virus completely. The need to improve patient care and to pursue new drugs discovery are important goals. However, they should not shadow the extraordinary advances achieved in outbreak response and mAb treatment that have fundamentally changed the prognosis of EVD from a near certainty of death to a potentially curable disease.

## Overview of FDA-Approved Medical Countermeasures Against EVD

### Development and Mechanism of Action

There are significant differences between mAb114 and REGN-EB3 in terms of how they were produced and how they work. (**Table 1**).

**Table 1:** Brief comparison of mAb114 and REGN-EB3 characteristics.

Features and properties	MAb114	REGN-EB3
**Presentation**	- Single mAb	- Cocktail of 3 mAbs (REGN3470, REGN3471, and REGN3479 in a ratio of 1:1:1)
**Origin**	- Derived from memory B cells from a survivor of the 1995 EBOV outbreak in Kikwit, Democratic Republic of the Congo, approximately 11 years after infection	- Immunization VelocImmune mice with DNA encoding Ebola virus glycoprotein and purified EBOVGP followed by cloning the human variable regions onto human constant regions, leading to a fully human antibody
**Targeted epitope**	- glycan cap and the core domain of the glycoprotein sub-unit 1 (GP1)	- REGN 3479: conserved GP2 fusion loop.
		- REGN 3471: outer glycan cap
		- REGN 3470: GP1 Head
**Mechanism of action**	- Neutralization	- Antibody-dependent cell cytotoxicity,
	- Antibody-dependent cell cytotoxicity	- phagocyte stimulants,
		- virus internalization inhibitors
**Advantages**	- Single Antibody	- Cocktail mAbs
	- Resisting at low pH environment (GP rearrangement with cathepsin);	- Targeting several different epitopes (reduce the selection of resistant virus)
	- Binding in a highly conserved region reduces the risk of escape mutants;	- Single shot
	- Single-shot option with short infusion time (30’ to 1h);	- Good half-life
	- Good half-life	
	- Highly stable	
	- Easy to manufacture with large scale production	
**Dosing**	- 50 mg/kg	- 150mg/kg

mAb114 is a human immunoglobulin G1 (IgG1) isolated 11 years after clinical infection of a 1995 Kikwit EVD survivor in DRC (19). mAb114 was selected after isolation and screening of a panel of memory B-cells for its ability to bind to the EBOV envelope glycoprotein (GP) and neutralize the virus *in vitro* (**Figure 5A**).

**Figure 1:**
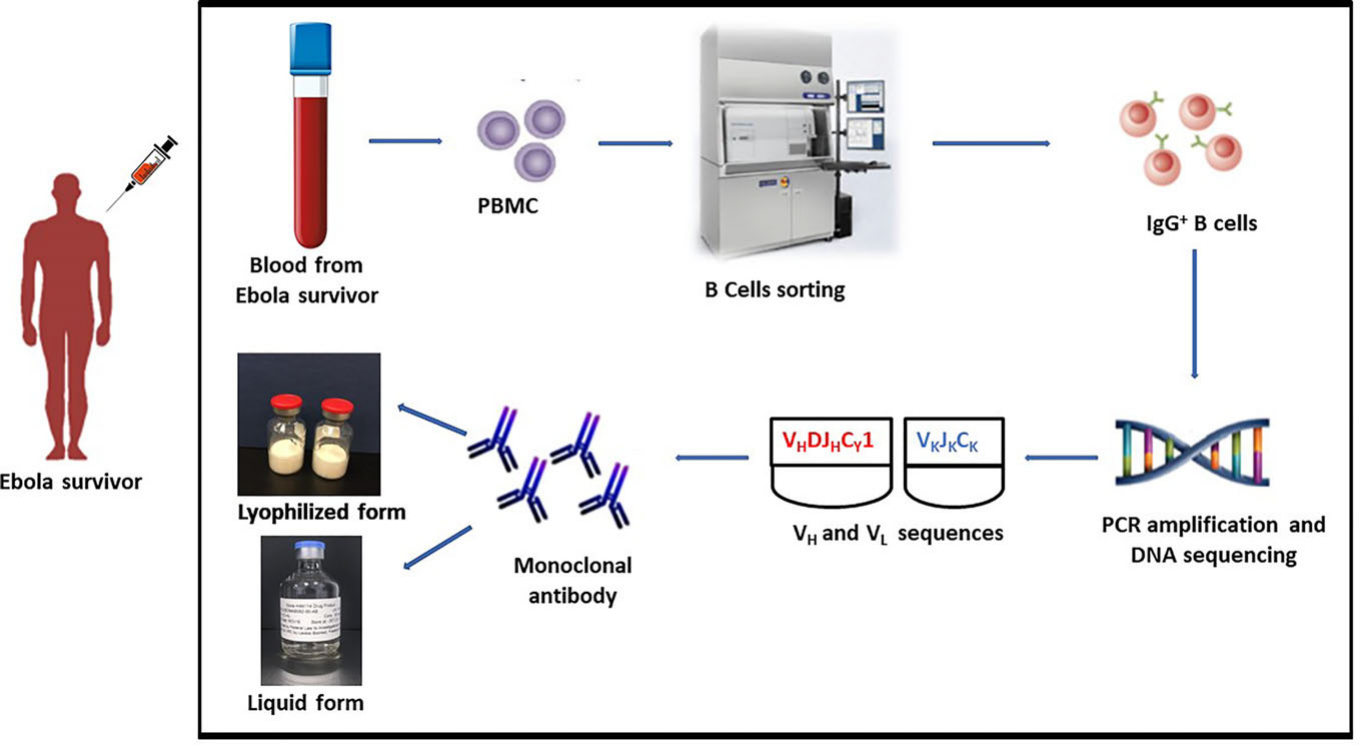
mAb114 development technique. Blood collected from EVD survivor. PBMC isolation followed by identification of the mAb114 memory B cell clone. Heavy and light chain sequences obtained by PCR amplification and DNA sequencing. Heavy and light chain sequences cloned into a standard expression vector. Expression vector used to generate a stably transfected CHO DG44 cell line for use in manufacture.

Heavy and light chain sequences were obtained by PCR amplification, and DNA sequencing after the mAb114 memory B cell clone was identified (**Figure 1**). A conventional expression vector was used to clone heavy and light chain sequences. This expression vector was utilized to produce a stable Chinese Hamster Ovary (CHO) DG44 cell line for manufacturing (19).

**Figure 2:**
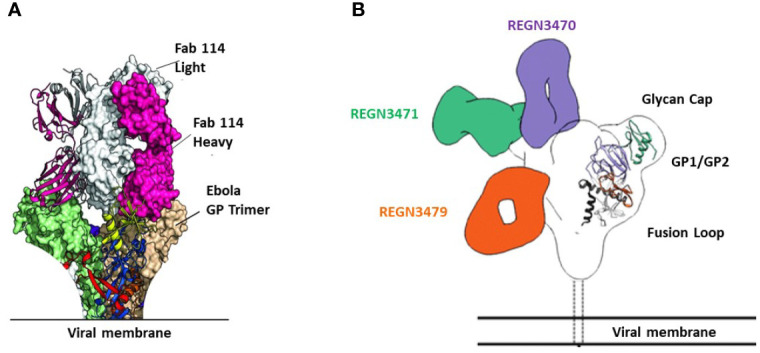
Binding sites of FDA-approved mAbs therapeutics on EBOV GP. **(A)** Crystal structure of GP Muc interacting with Fabs of mAb114. Fabs are shown in pink (heavy chain) and white (light chain). GP Muc promoters are in green and beige for molecular surfaces while the third is represented by ribbon strings. **(B)** Relative binding sites of REGN-EB3 on EBOV GP. adapted from ref (20). REGN3471, REGN3470, and REGN3479 combined reconstructions on a single EBOV GPΔTM, demonstrating the relative locations of the epitopes on GP from the three competition groups. Only one Fab is shown per antibody.

**Figure 3:**
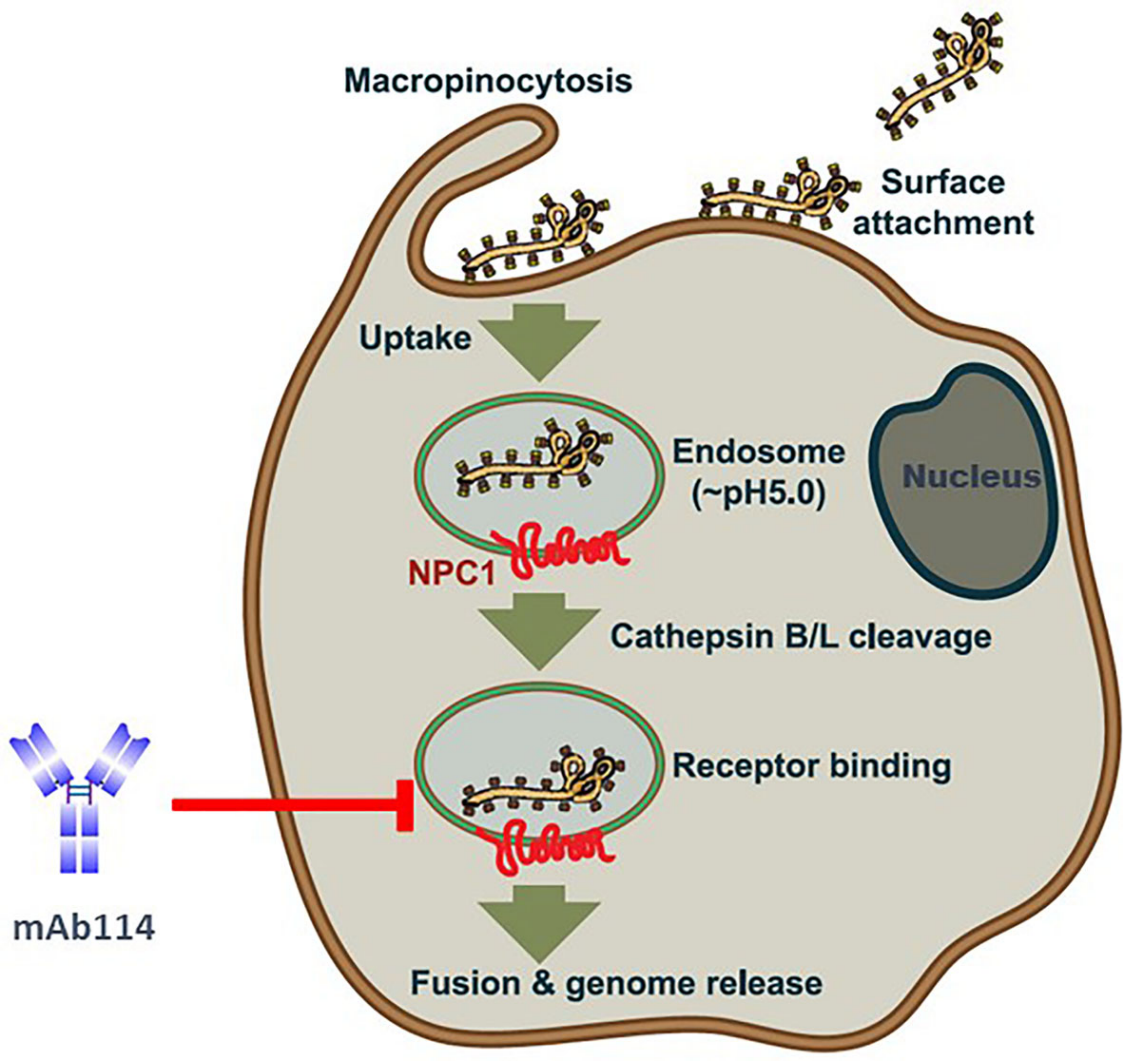
*Ebola virus* uptake, entry model, and mAb114 mode of action [(adapted from ref (21). Viral particles bind to various surface factors and trigger macropinocytosis, leading to trafficking to endosomes. Acid-dependent proteases (cathepsin B and cathepsin L) remove the MLD and glycan cap in the low-pH endosome environment, revealing the RBD in the GP1 core, previously blocked by these domains. The host cell receptor, NPC1(red), is then engaged by virions with exposed RBDs, resulting in the host and viral cell membranes fusion and the release of the viral genome into the target cell cytoplasm. In late endosomes, mAb114 binding prevents the GP from interacting with the host cell receptor protein NPC1. Brown shading indicates the cell membrane and cytoplasm. The endosomal membrane is green with a brown border, while the endosomal lumen is light gray. The nucleus is dark gray, with a blue-black membrane around it.

mAb114 targets the glycan cap and the core domain of the GP sub-unit 1 (GP1) of EBOV. This mAb binds to a conserved amino acid region on the receptor-binding domain (RBD) of the EBOV GP (**Figure 2A**) and remains bound both in physiological and low intracellular pH environments, preventing the GP from engaging the host cell receptor protein [Niemann– Pick intracellular cholesterol transporter 1 protein (NPC1)] in late endosomes (**Figure 3**).

The advantage of targeting the RBD is that it reduces the danger of escape mutants while preserving the mAb’s high neutralizing activity (19). Indeed, some viruses have evolved mechanisms that allow them to circumvent efficient neutralizing antibodies responses and instead trigger them at much lower doses. In the context of highly antigenic variable viral strains, effective responses refer not only to their neutralization potency but also to their ability to neutralize various circulating global isolates.

REGN-EB3 is a cocktail of 3 human IgG1 mAbs (REGN3470, REGN3471, and REGN3479) that has been identified as an effective cure against EBOV. These mAbs are of a similar canonical structure.

**Figure 4:**
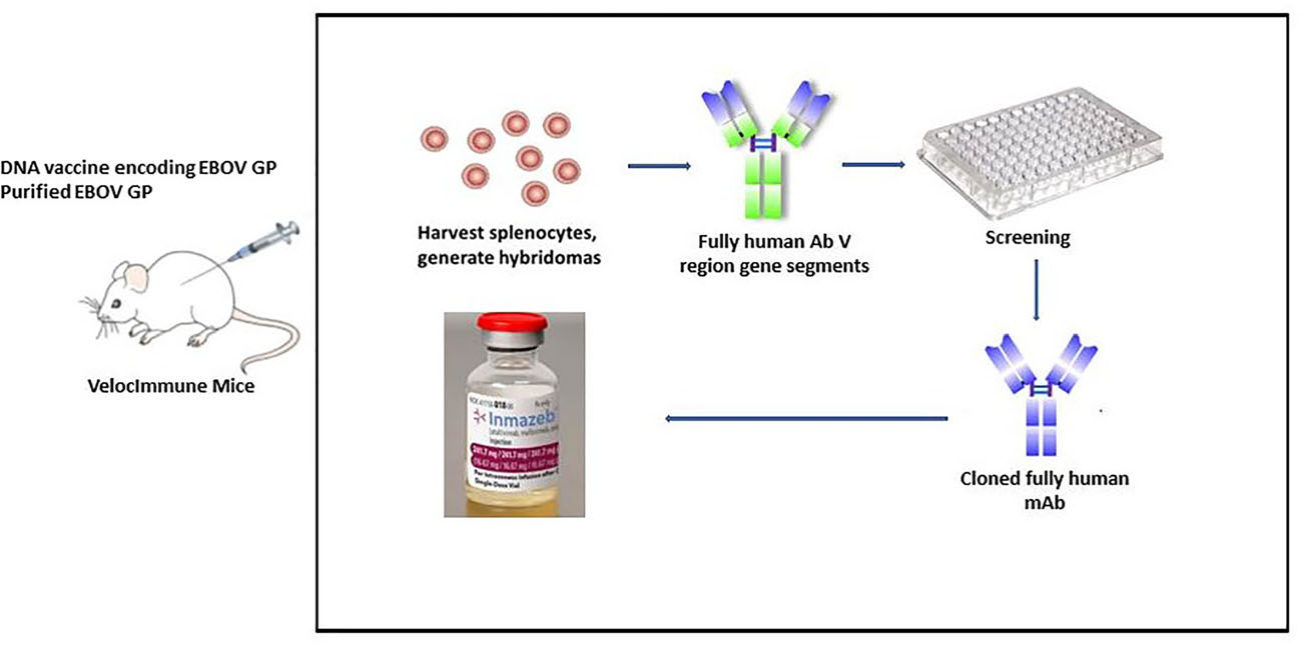
REGN-EB3 development technique. To generate an immune response, transgenic mice (VelocImmune) are immunized with a DNA vaccine encoding EBOV GP and/or recombinant EBOV GP. Splenocytes are harvested and fused with myeloma cells to create hybridoma cells that produce antibodies continuously. Selected leads are utilized to generate chimeric or humanized antibodies after they have been screened.

Immunizing mice (VelocImmune mice) with both DNA expressing EBOV GP and/or pure EBOV GP was the first step in developing REGN-EB3 (**Figure 4**). VelocImmune mice express human immunoglobulin heavy and kappa light chain variable regions and mouse constant regions. Mouse-generated antibodies were screened for their ability to block entry of EBOV GP-pseudotyped particles into susceptible cells. Additional tests were performed to evaluate binding to GP at endosomal pH, absence of cross-competition, binding to sGP, and Fc effector functions. Three mAbs (REGN3470, REGN3471, and REGN3479) were selected based on these screening criteria and then manufactured using CHO isogenic cell bank (20, 22).

**Figure 5:**
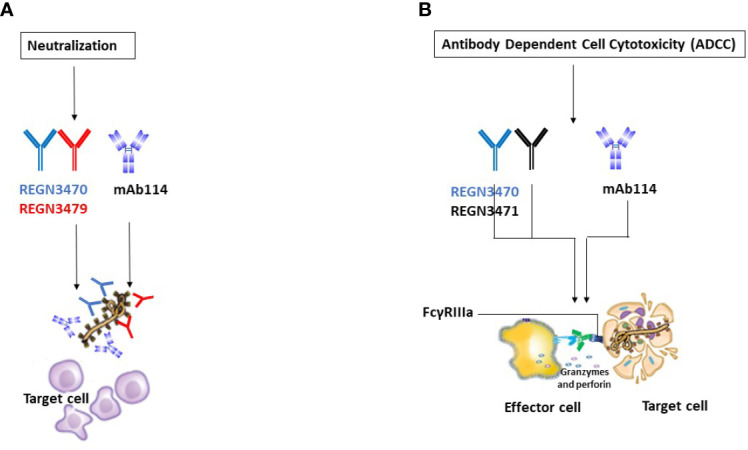
Illustrative mechanisms of FDA-approved mAbs therapeutics. **(A)** Neutralization Illustrative representation of mAb114 (lavender color) and two REGN-EB3 components [REGN3470 (blue color) and REGN3479 (red color)] neutralization activity. **(B)** ADCC. Illustrative representation of mAb114 (lavender color) and two REGN-EB3 components [REGN3470 (blue color) and REGN3471 (black color)] ADCC activity.

The three antibodies in REGN-EB3 bind to non-overlapping epitopes on the GP, (**Figure 2B**) potentially increasing efficacy and reducing the likelihood of escape mutants (22). The complementarity-determining regions within the heavy chain and light chain variable domains of each mAb combine to form the binding site for its target, the GP protein of EBOV (22). REGN3479 has neutralizing activity and prevents the entry of the virus into the host cell (**Figure 5A**). Upon binding to its target, REGN3471 engages FcγRIIIa to trigger ADCC function. REGN3470 combines neutralization and ADCC activities (**Figure 5B**). Each mAb is produced in low fucose cell lines to enhance binding to FcγRIIIa and effector function activity (20).

### Preclinical Experience

mAb114’s therapeutic potential has been tested in Non-Human Primates (NHPs) in EBOV challenge experiments. These investigations found that a single 50 mg/kg or 30 mg/kg infusion given up to 5 days after the challenge provided complete protection from death, indicating that a single dosage of mAb114 could be a viable treatment for EBOV (23).

The repeated dose Pharmacokinetic (PK) of mAb114 was characterized using healthy rhesus monkeys (23). mAb114 was administered over four weeks by IV administration either once weekly at dose levels of 0 (vehicle control), 50, and 500 mg/kg or thrice weekly at a dose level of 50 mg/kg (150 mg/kg/week). Serum samples were collected pre-dose on Day 1 and at post-dose time points throughout the dosing phase of the study and 8-week recovery for quantitating serum levels of mAb114 and characterization of PK. The studies found no detectable levels of mAb114 in the pre-dose samples or the samples taken from the control group (0 mg/kg IV). After IV administration, the maximal blood concentrations of mAb114 were frequently attained 15 to 60 minutes post-dose for both sexes, following the first and last doses. The increase in plasmatic concentration between 50 and 500 mg/kg/week was generally dose-proportional for both sexes.

In summary, PK analyses revealed that substantial levels of mAb114 in blood were achieved in monkeys after IV administration, with no significant gender-related changes in PK characteristics. According to the findings, mAb114 has linear kinetics, accumulates after the repeated dose, and most monkeys were exposed for up to 77 days. The clearance of mAb114 was defined, with mean terminal half-lives ranging from 7 to 15 days when they combined t1/2 data from both sexes (19).

EBOV challenge tests in NHP have also been used to assess REGN-EB3’s therapeutic potential (20). NHPs were given the mAb cocktail to determine efficacy, define the effective dose and the frequency of administration. These studies found that administration of antibody cocktail at different dose-regimens post-infection with the Kikwit strain of the virus can treat an established EBOV infection and help animals recover from advanced EVD.

Animals given 150mg/kg of mAb cocktail demonstrated a better survival rate than placebo. However, survival is dosage-dependent, and for maximum therapeutic benefit, a single dose of 100 mg/kg or higher was necessary (20).

REGN-EB3 PK data on NHPs are still unpublished, but they served to guide development of PK experiment during the phase 1a clinical study (22). The trial studied four dose levels (3, 15, 60, and 150 mg/kg). The PK of REGN-EB3 following intravenous (IV) infusion was linear, and t1/2 was dose-independent for each mAb and ranged from 20.2 to 22.7 days, the t1/2 for REGN-EB3 was in the range expected for a typical mAb exhibiting linear PK (22).

### Clinical Experience

mAb114 and REGN-EB3 were used in adult and pediatric subjects infected with EBOV in the PALM RCT and under EAP during the 2018 EBOV outbreak in DRC. The efficacy and safety results of these two therapeutics are summarized in the previous section of this review. During subsequent EBOV outbreaks, notably in the provinces of Equateur/DRC in 2020, North-Kivu/DRC in 2021, and N’Zerekore/Guinea in 2021, the two therapeutic mAbs were also used for the treatment of EVD patients under the EAP.

mAb114 is available in two injectable forms: a lyophilized form stored between 2°C and 8°C and a frozen liquid form stored between -35°C and -15°C. mAb114 recommended dose is 50 mg/kg administered as a single IV infusion over 60 minutes after dilution in 0.9% Sodium Chloride Injection or Lactate Ringer. The lyophilized product is first reconstituted with Sterile Water for Injection before further dilution.

REGN-EB3 is delivered as a sterile aqueous solution to store in the refrigerator between 2°C and 8°C. REGN-EB3 is administered as a single IV infusion following dilution in 0.9% Sodium Chloride Injection, Lactate Ringer Injection, or 5% Dextrose Injection at a dose of 150 mg/kg (50 mg of REGN3470, 50 mg of REGN3471, and 50 mg of REGN3479).

### Current Scientific Questions Related to Ebola-Specific mAbs Use

Due to the fast evolution of viral infections, the risk of developing drug resistance is a problem for any mAb therapy. When selective pressure is applied in the context of medication therapy, such resistance becomes more apparent. To achieve better efficacy and to prevent viral escape mutation, a cocktail of two or more mAbs is preferred over a single mAb. Still, this approach increases manufacturing volumes and costs, complicates formulation (24, 25), and prevents novel strategies like antibody delivery *via* viral vectors or non-vectored nucleic acids (26). Since the single mAb KZ52 failed to confer complete protection against EBOV in NHPs in 1999 (27), single mAb therapy was considered a risky approach. Recently, mAb114 has changed this therapeutic consideration in the Ebola field. Nonetheless, a recent report mentioned a relapse case of acute EVD in a survivor of the 2018–2020 EBOV outbreak in North-Kivu and Ituri provinces in the DRC, previously treated with mAb114 (28). This observation brought back the question related to virus mutation escaping single mAb therapeutic pressure, resulting in variant harboring resistance. Investigations performed on the isolated variant virus showed the distant location of mutations on viral envelop GP (G6800A/E258K and A6867G/E280G) from mAb114 binding site (29). Furthermore, *in vitro* assays performed to test the ability of mAb114 to neutralize viruses bearing the mutant GP (30) excluded mAb114 selective pressures hypothesis as a potential cause of the virus GP variation. However, considering the unpredictable occurrence of mutations resulting from an adaptive evolutionary process, improvement of mAb114 to develop the ability to bind at more than one epitope could mitigate the potential risk of inducing virus mutation. This improvement will build on the rationale behind the development of mAb114, which is to provide a simplified therapeutic product and dosing regimen for EVD, identify the mechanism(s) of EVD protection, and explore development for potential stockpiling.

Bispecific antibodies have recently attracted a lot of attention since they allow for unique mechanisms of action and therapeutic uses that are not possible with traditional IgG-based antibodies.

Klein et al. (31) developed the CrossMab technology, that uses domain crossover of Ig domains in the bispecific antibody’s Fab region to ensure proper light chain interaction (25). CrossMab technology has demonstrated promising results in animal models to treat various viral diseases such as Zika and COVID-19 (29, 30). To date, only a few studies have explored the proof-of-concept of this technique for Ebola, using small animal models (32–34). The therapeutic synergy of bispecific mAbs has not yet explicitly been demonstrated, and effectiveness also needs to be shown in large animal models to allow progression to clinical trial.

The PALM RCT used a defined mAb114 dose of 50mg/kg, having demonstrated a linear PK in phase 1 clinical trial with a half-life of 24.2 days (19). In most cases, systemically administered mAbs have biphasic PK patterns in circulation, with a quick distribution and a later elimination phase. Because of their size and solubility, mAbs have a limited distribution in the vasculature and interstitial space, as well as lengthy half-lives (11–30 days in humans). Fundamental PK parameters of mAbs such as clearance and half-life are dose-dependent and nonlinear clearance is expected and often observed at low doses. Linear and predictable clearance is expected above the saturation dose range (35). One hypothesis is that the current mAb114 dose not reaching saturation and could explain some therapeutic failures in patients with high viral load. Using a higher dose of mAb114 (>50mg/kg) to reach saturation may be considered to improve product bioavailability and patient survival.

Other functional properties of mAb114 need to be better explored to understand all mechanisms involved in protecting against the virus. Corti et al. (23) speculated that the mAb114 antibody’s particular neutralization mechanism and *in vitro* ADCC (**Figure 5B**) activity might contribute to its ability to protect macaques from deadly EVD. The production of ADCC-inducing antibodies or the addition of ADCC-enhancing mutations to neutralizing antibodies, according to Liu et al. (36), could improve their preventive or therapeutic capability against EBOV infection. Engagement of FcɣRs is required for antibody effector functions (37).

Point mutations, glycoengineering, Fc domain exchange, exchange across isotypes, and IgG multimerization affect binding to FcɣRs, resulting in increased ADCC (38). These modifications may be envisaged to improve mAb114’s effector functions.

The cocktail REGN-EB3 seems to present great benefice for the treatment of EVD patients, but its manufacturing for large stockpile production may be costly (production of three mAbs) (39). Potential concerns related to product saturation may also be applied for REGN-EB3 administered to deceased patients with a high viral load during the PALM RCT study. Room for improvement is still available, and strategies used to increase the potency of any mAb could also find its application with REGN-EB3 for enhancing the therapeutic effect of the product.

The efficacy of both mAb products is noticeable. However, it is worth exploring the cost-effectiveness, the number of doses that can be manufactured, and the possible target populations for use. Improvement on both products should be prioritized for future use.

## Putative Role of EBOV Soluble Glycoprotein as a Decoy of the Immune System and Treatment Failure

**Figure 6:**
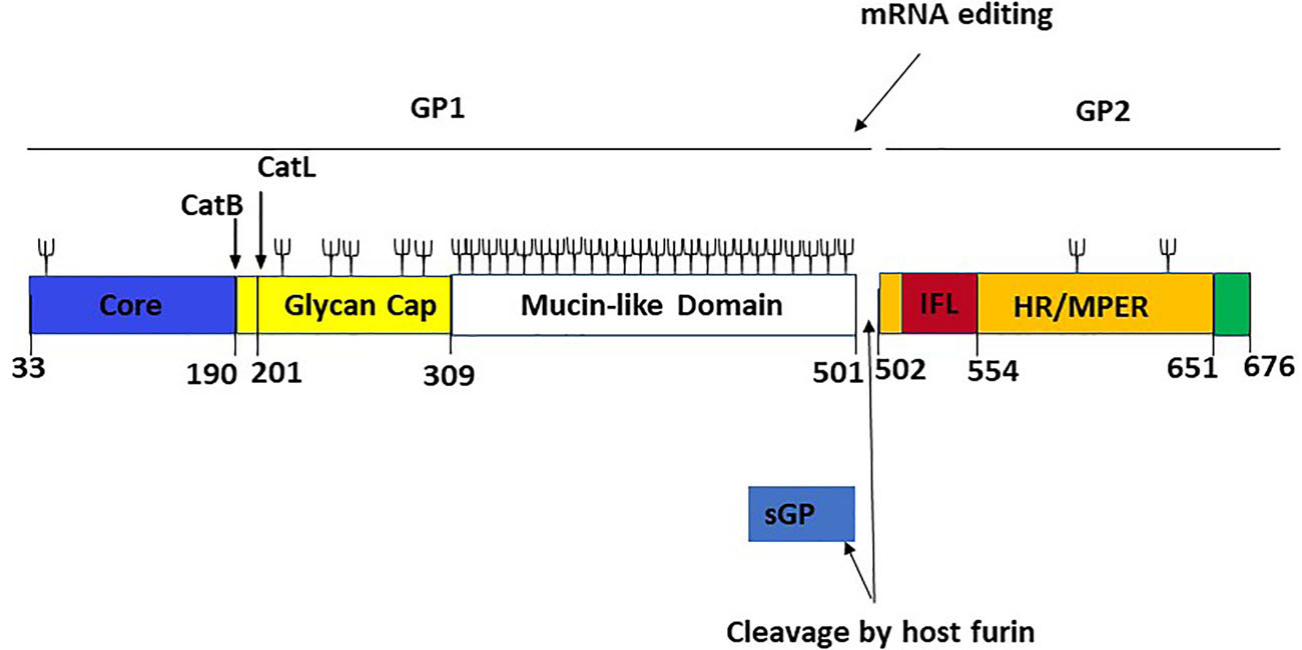
Linear schematic representation of the *Ebola virus* GP monomer with GP1,2 subunits and sGP [adapted from ref (21)]. The GP1 core (blue, residues 33–189), the cathepsin cleavage loop (yellow, residues 190–210), the glycan cap (yellow, residues 211–308), and the highly glycosylated mucin-like domain (white, residues 309–501) are the four parts of GP1. An internal fusion loop (IFL) region (red, residues 502–553), an hepta repeat (HR)/membrane-proximal external region (MPER) (orange, residues 554–650), and a transmembrane domain/cytoplasmic tail region (green residues 651–676) constitute GP2. The co-transcriptional editing of the GP gene-editing site, which results in the transcript of the sGP product and the post-transcriptional cleavage of the GP precursor by a host furin, are also shown. The proteolytic cleavages of cathepsin B (CatB) and cathepsin L (CatL) are also depicted. Cathepsin B cleaves at position 190, while cathepsin L cleaves at position 201. Branched lines indicate the approximate positions of potential glycosylation sites.

Understanding how EBOV suppresses or evades the host immune response is crucial for developing vaccines and MCMs against the virus. The release of a shortened viral glycoprotein, sGP, by EBOV-infected cells is one widely postulated immune evasion strategy used by EBOV. sGP is a non-structural protein resulting from transcriptional editing of the native GP (**Figure 6**). GP1 and GP2 are the major structural components found on the EBOV surface, and they mediate host cell adhesion and fusion. As a result, most EBOV vaccine and treatment research focuses on GP1,2, and it is widely believed that robust anti-GP1,2 antibody response is required for protection against deadly EBOV infection (40). Also, the binding of mAbs on sGP is not required to confer protection against EBOV (41). Like influenza Hemagglutinin (HA) and HIV Envelop (Env) protein, EBOV GP1,2 produces trimeric spikes on virion surfaces (42). GP is a Class I fusion protein, like HA and Env. GP is produced as an uncleaved precursor (GP0), which is then cleaved into two functional subunits in the Golgi complex by the protease furin (43). The N-terminal GP1 subunit contains the putative RBD, while the C-terminal GP2 subunit contains the fusion apparatus and transmembrane domain. The virus genome encodes GP1,2 in two fragmented reading frames.

The two reading frames are linked by the viral polymerase inserting an adenosine at an editing site (a tract of 7-As), resulting in an mRNA transcript that facilitates the read-through translation of GP1,2 (44, 45). However, only about 20% of transcripts are edited, leaving the remaining 80% with a premature stop codon, resulting in the production of a truncated glycoprotein product that is discharged in large quantities into the extracellular space. Although all EBOV species produce sGP, its role has been a source of controversy. Unlike GP1,2, sGP forms homodimers and appears to have some intrinsic anti-inflammatory activity (46, 47).

The finding that EBOV rapidly mutates to synthesize mostly GP1,2 in cell culture but reverts to a largely sGP-producing phenotype *in vivo* shows that sGP plays a significant role in virus survival within the host (48). According to the existing evidence, sGP could be engaged in various processes ranging from repairing endothelial cell barrier capabilities to influencing the immunological response (49). Because more than 90% of sGP’s sequence overlaps with the N-terminal portion of GP1,2, it was first thought that sGP could act as a decoy for anti-GP1,2 antibodies. Early attempts to find such activity produced conflicting results, and placed doubt on the concept (50–54). Furthermore, recent research has shown that immunizing against GP1,2 generates antibodies mostly against epitopes that are not shared with sGP (55–58). However, the majority of these researches looked at mAbs from animals inoculated with vaccines that contained or expressed predominantly GP1,2, which does not reflect the state of infection in nature. One recent study looked at mAbs from mice vaccinated with a Venezuelan equine encephalitis replicon that generates both GP1,2 and sGP and discovered that many of these antibodies cross-reacted (59). Additionally, human EVD survivors’ mAbs have been demonstrated to react with sGP (50).

During the RCT PALM study, FDA-approved EVD therapeutics demonstrated significant efficacy in reducing EVD mortality. Nevertheless, residual mortality of 34% and 35%, respectively for REGN-EB3 and mAb114, has been reported for patients randomized under these products. With its role as a decoy, sGP could be one of the potential reasons for residual mortality by binding EBOV-neutralizing antibodies and impairing a protective humoral immune response (52, 60). Evidence has shown that mAb114 cross-reacts with sGP (23) and one of the mAb included in the REGN-EB3 cocktail also cross-reacts with sGP (20).

The significant degree of similarity between sGP and GP suggests that passive immunotherapies aimed at GP may interact with sGP (21). The protomers of sGP adopt a conformation similar to that of GP’s overlapping area, which comprises the whole GP1 core and nearly the whole glycan cap region (21). Analysis of samples from dead patients gathered during the study could be an appealing way to establish the role of sGP as an immune decoy and could be useful in developing new therapies or vaccine methods.

## Hypothetical Risk of mAbs Administration to Early Events of Downstream Immunity Development in EVD Patient

**Figure 7:**
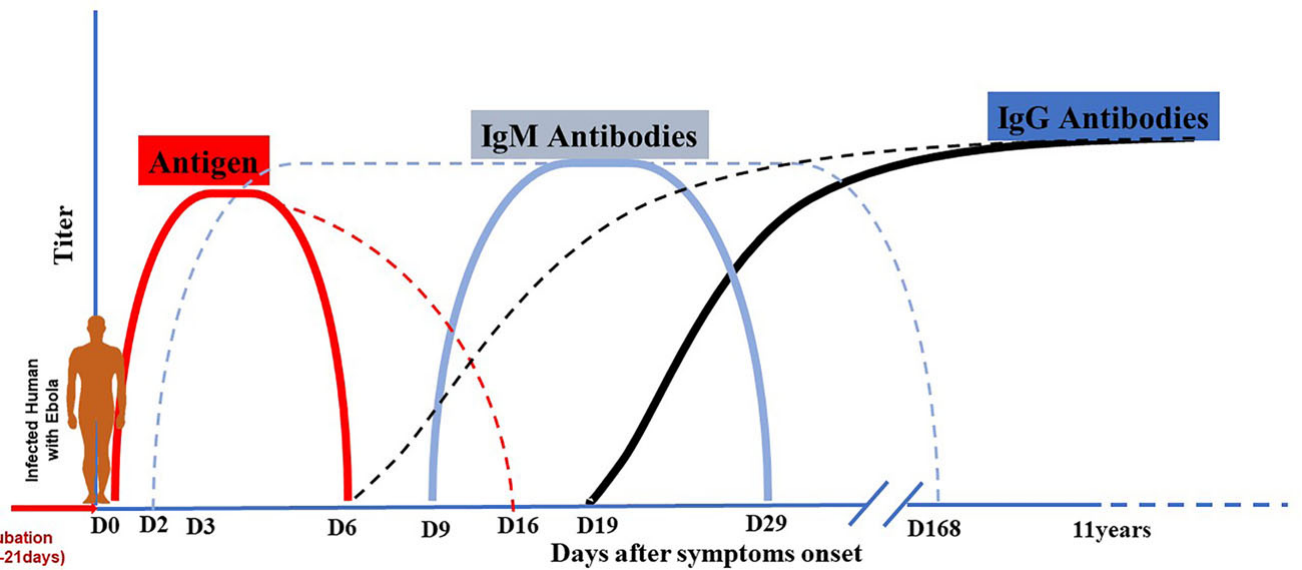
Antigen and antibodies kinetic during EBOV infection. Dotted lines indicate maximal range, and solid lines indicate mean range. After the period of incubation estimated from 2 to 21 days, EVD patients become symptomatic. Virus antigen becomes detectable early after the beginning of symptoms and can persist until day 6-16. Antibody production kinetic during EVD is described as an early occurrence between day 2 and 9 for IgM and between day 6 and 19 for IgG. IgM quickly reach the plateau and fall off around day 29 or longer (day 168); while IgG increase progressively to reach the plateau and last for years (11-40 years).

The balance between virus and host defenses determines the outcome of each step of viral infection, from viral entry to local replication, dissemination (viremia), amplification in targeted organs, the onset of disease, and finally recovery (virus shedding) or death. Local (innate immunity) and systemic (specific humoral and cellular immunity) recovery mechanisms are triggered at each stage of virus propagation through the body. The uncontrolled viral replication of EBOV is fundamental to its pathogenesis because of cytopathic effects and deregulation of the host immune response. During EVD, virally driven immune system damage develops through several pathways. Furthermore, it is thought that the Ebola virus predominantly targets macrophages and dendritic cells, resulting in a weakened innate and adaptive immune response, allowing for uncontrolled viral replication and spread (36, 61). Patients recovering from EVD develop antibodies that can persist for several years in the plasma. Antibody production kinetics during EVD is described as an early occurrence between day 2 and 9 for IgM and between day 6 and 19 for IgGs. IgMs quickly reach the plateau and fall off around day 29, while IgGs progressively reach the plateau and lasts for years (**Figure 7**) (62, 63). This kinetic profile is similar to what is observed in some viral infections. For infected subjects who mount a robust immunity and successfully control infection, antibodies developed can confer lifelong protection against future exposure (64).

Concerns have been raised in the field about the potential effect of early EBOV-specific therapeutics (especially in the setting of mild or subclinical disease) on downstream protective immunity, given the recent introduction of FDA-approved therapeutics in medical care management and its potential large rollout during future outbreaks. This hypothetical concern is essential to investigate and may provide valuable contributions in the field management of survivors as exemplified during the 2018-2020 EVD outbreak in North-Kivu and Ituri provinces in DRC. The approach consisted of involving cured patients or survivors (HCW and non-HCW) in playing integrated roles in the care of EVD patients inside the ETC after discharge. Typically, ETCs utilizing this strategy have deployed EVD survivors with “light PPE” for contact precautions rather than the full EVD PPE. This strategy has allowed them to stay inside the ETC “red zone” for more extended periods and provide constant care for the most vulnerable patients, including children, the elderly, and pregnant women. Indeed, their role as “guardians of the sick” has importantly enabled the optimized supportive and psychological care in this outbreak, particularly by increasing patient monitoring and providing comfort to these fragile populations. The underlying assumption is that EVD survivors would generally have protective immunity to typical exposures to the same outbreak isolate and would not be re-infected with EBOV. However, protective immunity in the human survivor population has not yet been well characterized, especially in an EVD-specific treated cohort. In addition, the effect of EVD-specific treatment on an individual ability to mount a robust immune response has not yet been investigated. Administration of mAbs in EVD patients during the disease may mitigate early events involved in antigenic exposure to the immune system. However, it could jeopardize the subsequent development of robust natural immune response during future EBOV exposure. It is therefore critical that we thoroughly study the adaptive immunity in this growing cohort of treated survivors.

## Potential Interactions Between Prior Vaccination With FDA-Approved EVD-Specific Vaccine and Therapeutics

The US FDA licensed the first vaccine against EBOV in 2019, a recombinant, live-attenuated viral vector in which the G protein of vesicular stomatitis virus is replaced with the GP of EBOV (rVSV-EBOV-GP, Ervebo^®^ by Merck) (65). This vaccine provided preventive, immediate, and post-exposure protection in NHPs, demonstrating significant efficacy.

During the 2013–2016 West Africa outbreak, rVSV-EBOV-GP showed 100% human protection in many phase III clinical studies involving over 10,000 people (66).

Following the widespread use of rVSV-EBOV-GP in the North-Kivu EVD outbreak, more than 218,000 doses were given to people at high risk of EBOV exposure by 2020 (67). In addition, three MCMs were tested against a comparator arm, Zmapp, in the first EVD RCT, PALM, conducted during the same outbreak. It was the first time that investigational anti-EVD drugs were widely employed, resulting in the largest cohort of EVD patients ever treated.

However, no detailed immunological description of vaccination or MCMs-elicited immunity from this cohort has been established to our knowledge. Furthermore, few investigations in West Africa have attempted to define the immunological profile of survivors, including vaccinated subjects (68), without considering the possibility of interaction between the two therapies. Also, the delay in introducing those therapeutics at the tail of the epidemic in West Africa made it challenging to assemble reliable and conclusive data.

The PALM study also recorded participants’ vaccination status based on self-reported. Even though the source of vaccination status may present a limitation, 25% of all PALM enrolled participants reported being vaccinated prior to the onset of symptoms (15). While the rVSV-EBOV vaccine has very high efficacy, breakthrough infections still occur, possibly due to insufficient vaccine-induced immunity (69). Given the likelihood of future widespread use of this vaccine in hot zones during outbreaks and preventive administration to frontline healthcare workers, close attention needs to be provided to the vaccine-elicited immunity to avoid putting vaccinated individuals at risk.

The utilization of EBOV-specific therapeutics during the susceptibility windows post-vaccination has prompted doubts and concerns about agonist or antagonist effects between mAbs-based therapeutics and the vaccine. The most serious worry is the possible harmful interaction caused by co-administration of an EBOV GP vaccine with therapeutic mAbs that target EBOV GP, which is currently the most effective post-exposure EBOV vaccine for humans. Previous preclinical research indicated that vaccinating animals before the EBOV challenge and then treating them afterward provided complete protection without any clinical illness (70). Furthermore, results from self-reported vaccination status collected during the PALM study revealed a low mortality rate among infected vaccinated participants as compared to those who reported no vaccination (27.1% *vs*. 48.4%) (15), which support a benefice of mAbs-based therapy and vaccine combination as demonstrated by the experience of T.W Geisbert and team on NHPs (70). However, additional investigations need to be done to confirm the vaccination status of those PALM participants. Sequential human samples need to be collected from future outbreaks to corroborate this finding, though this will be challenging given the unpredictable occurrence and remote location of Ebola outbreaks.

Therefore, it is understood that breakthrough infection may occur in vaccine recipients. Thus, the treatment option should consider addressing the need for further investigations to deeply understand current FDA-approved vaccine and therapeutic interactions.

## The Development of Pan-Filoviruses mAbs and mAbs Against Other Species of *Ebolavirus* Remains Vital for Protection

EBOV, SUDV, BDBV, TAFV, RESTV, and the newly described BOMV are the six *Ebolavirus* species that have been isolated so far. While RESTV does not cause disease in humans and TAFV has only been linked to one nonlethal infection, EBOV and SUDV have been linked to significant human EVD epidemics with CFRs ranging from 25 to 90% (71). In the NHP model of *Ebolavirus* infection, all five classical *Ebolavirus* species are fatal (71). EBOV and related filoviruses (SUDV and *Marburgvirus*) have produced periodic epidemics since their discovery in 1976, each impacting a few hundred persons within a geographically confined area. While the outbreaks were concerning, no evidence of filovirus spreading as a massive epidemic through urban and rural regions was discovered until 2013 (72). The 2013 Ebola outbreak and the newly evolved severe acute respiratory syndrome coronavirus 2, responsible for the current coronavirus disease pandemic since 2019 (73) illustrate that emerging and re-emerging viruses pose a health concern to humans.

It is then expected that other filoviruses with the potential to cause an outbreak with severe disease manifestation will emerge; we should be prepared with prevention and therapeutics tools to intervene in such an occurrence. Based on the success of two mAb-based therapies against EBOV in clinical trials (15) and their approval by the US FDA for the treatment of EVD in adults and pediatric patients (74), research should focus on the development of mAbs capable of neutralizing multiple filoviruses and pan-species *Ebolavirus* mAbs. Crowe and team isolated one mAb from human survivors of EVD which bound to an epitope in the GP base region and cross- neutralized EBOV, BDBV, and SUDV in small model animal (75). Bornholdt et al. isolated two broadly neutralizing human antibodies which when formulated as a cocktail (MBP134AF) could protect ferrets and NHPs against lethal EBOV, SUDV and BDBV (41). Holtsberg et al. developed pan-ebolavirus and pan-filovirus antibodies which cross-neutralized EBOV and SUDV *in vivo* (76). All those studies exemplify the ongoing efforts towards the development of broadly neutralizing mAbs, pan-ebolavirus and pan-filovirus antibodies. However, cross-species neutralizing mAbs identified need to be fully pre-clinically characterized and enter into an accelerated development path towards clinical trials. Current antibody engineering techniques may help to improve their functionality and efficacy, as demonstrated by Frei et al. in small model animals (32, 33). With the perspective to be effective against any all-known *filoviruses* infection, the same effort must be considered for the fast development of pan-filoviruses mAbs.

## Conclusion

Antibody-based immunotherapy is an effective intervention against emerging viral infections. Although some general features of mAb114 and REGN-EB3 differ, as outlined in this review, their initial implementation has proved efficacy in significantly reducing EBOV mortality. Antibody-based treatment to EVD will need to be monitored for rapidly evolving virus resistance. Usage of a cocktail of mAbs, such as REGN-EB3, targeting non-overlapping epitopes of EBOV should reduce the risk of selection of resistance. To improve efficacy and reduce the possibility of resistance, a single EBOV-specific mAb, such as mAb114, can be engineered with a mix of specificities to distinct epitopes. Several burning questions in the Ebola field related to the introduction of mAbs therapeutic have been discussed in this review and still need more investigations to be elucidated. Future research must focus on resolving those questions and enhancing progress made after over 44 years of extensive research on Ebola therapeutics. Also, the current FDA-approved therapeutics constitute an essential milestone in the Ebola therapeutic research and may serve as a backbone for designing novel treatments or vaccine strategies.

## Author Contributions

OTM contributed to the selection of the articles, redaction, and editing of the review. PM contributed to redaction and editing of the review. SM contributed to the editing of the review. All authors contributed to the article and approved the submitted version.

## Funding

Research Center on Infectious Diseases at Université Laval, Quebec, Canada.

## Conflict of Interest

OTM is employed by Leidos Biomedical Research. SM is employed by Ridgeback Biotherapeutics, and is listed as inventor on the patent application for mAb 114, US Application No.62/087, 087 (PCT Application No. PCT/US2015/060733) related to anti-Ebola virus antibodies and their use.

The remaining authors declare that the research was conducted in the absence of any commercial or financial relationships that could be construed as a potential conflict of interest.

## Publisher’s Note

All claims expressed in this article are solely those of the authors and do not necessarily represent those of their affiliated organizations, or those of the publisher, the editors and the reviewers. Any product that may be evaluated in this article, or claim that may be made by its manufacturer, is not guaranteed or endorsed by the publisher.

## References

[1] Bres P. The Epidemic of Ebola Haemorrhagic Fever in Sudan and Zaire, 1976: Introductory Note. Bull World Health Organ (1978) 56(2):245. 307454 PMC2395566

[2] Burke J Declerq R Ghysebrechts G. Ebola Haemorrhagic Fever in Zaire, 1976. Report of an International Commission. Bull World Health Organ (1978) 56(2):271–93. PMC2395567 307456

[3] Deng IM. Ebola Haemorragic Fever in Sudan, 1976. Bull World Health Organ (1978) 56(2):247–70. PMC2395561 307455

[4] CDC. History of Ebola Virus Disease (EVD) Outbreaks Error Processing SSI File (2021). Available at: https://www.cdc.gov/vhf/ebola/history/chronology.html.

[5] Malvy D McElroy AK de Clerck H Günther S van Griensven J. Ebola Virus Disease. Lancet (2019) 393(10174):936–48. 10.1016/S0140-6736(18)33132-5 30777297

[6] Zhang L Wang H. Forty Years of the War Against Ebola. J Zhejiang Univ Sci B (2014) 15(9):761–5. 10.1631/jzus.B1400222 PMC4162877 25183030

[7] Saphire EO. An Update on the Use of Antibodies Against the Filoviruses. Immunotherapy (2013) 5(11):1221–33. 10.2217/imt.13.124 PMC4465755 24188676

[8] Mupapa K Massamba M Kibadi K Kuvula K Bwaka A Kipasa M. Treatment of Ebola Hemorrhagic Fever With Blood Transfusions From Convalescent Patients. J Infect Dis (1999) 179(SUPPL. 1):18–23. 10.1086/514298 9988160

[9] WHO Interim Guidance. Potential Ebola Therapies and Vaccines (2014). Available at: https://apps.who.int/iris/bitstream/handle/10665/137590/WHO_EVD_HIS_EMP_14.1_eng.pdf.

[10] The PREVAIL II Writing Group. A Randomized, Controlled Trial of ZMapp for Ebola Virus. N Engl J Med (2016) 375(5):1448–56. 10.1056/NEJMoa1604330 PMC5086427 27732819

[11] WHO. Ebola Virus Disease | WHO | Regional Office for Africa (2021). Available at: https://www.afro.who.int/health-topics/ebola-virus-disease.

[12] WHO. (2021). Available at: https://www.who.int/csr/resources/publications/ebola/ebola-ring-vaccination-results-12-april-2019.pdf.

[13] Nag A Chowdhury RR. Piperine, an Alkaloid of Black Pepper Seeds can Effectively Inhibit the Antiviral Enzymes of Dengue and Ebola Viruses, an *in Silico* Molecular Docking Study. Virus Dis [Internet] (2020) 31(3):308–15. 10.1007/s13337-020-00619-6 PMC7458978 32904842

[14] WHO. (2021). Available at: https://apps.who.int/iris/bitstream/handle/10665/326111/SITREP_EVD_DRC_20190728-eng.pdf.

[15] Mulangu S Dodd LE Davey RT Tshiani Mbaya O Proschan M Mukadi D. A Randomized, Controlled Trial of Ebola Virus Disease Therapeutics. N Engl J Med (2019) 381(24):2293–303. 10.1056/NEJMoa1910993 PMC10680050 31774950

[16] Davey R Fowler R Guenther S Muyembe J-J Carson G Mulangu S. Notes for the Record: Consultation on Monitored Emergency Use of Unregistered and Investigational Interventions (MEURI) for Ebola Virus Disease (EVD). World Heal Organ (2018) 1(August):1–7.

[17] FDA. FDA Approves First Treatment for Ebola Virus (2020). Available at: https://www.fda.gov/news-events/press-announcements/fda-approves-first-treatment-ebola-virus.

[18] FDA. FDA Approves Treatment for Ebola Virus (2021). Available at: https://www.fda.gov/drugs/drug-safety-and-availability/fda-approves-treatment-ebola-virus.

[19] Gaudinski MR Coates EE Novik L Widge A Houser KV Burch E. Safety, Tolerability, Pharmacokinetics, and Immunogenicity of the Therapeutic Monoclonal Antibody Mab114 Targeting Ebola Virus Glycoprotein (VRC 608): An Open-Label Phase 1 Study. Lancet (2019) 393(10174):889–98. 10.1016/S0140-6736(19)30036-4 PMC6436835 30686586

[20] Pascal KE Dudgeon D Trefry JC Anantpadma M Sakurai Y Murin CD. Development of Clinical-Stage Human Monoclonal Antibodies That Treat Advanced Ebola Virus Disease in Nonhuman Primates. J Infect Dis (2018) 218(Suppl 5):S612–26. 10.1093/infdis/jiy285 PMC6249601 29860496

[21] Misasi J Sullivan NJ. Immunotherapeutic Strategies to Target Vulnerabilities in the Ebolavirus Glycoprotein. Immun [Internet] (2021) 54(3):412–36. 10.1016/j.immuni.2021.01.015 33691133

[22] Sivapalasingam S Kamal M Slim R Hosain R Shao W Stoltz R. Safety, Pharmacokinetics, and Immunogenicity of a Co-Formulated Cocktail of Three Human Monoclonal Antibodies Targeting Ebola Virus Glycoprotein in Healthy Adults: A Randomised, First-in-Human Phase 1 Study. Lancet Infect Dis [Internet] (2018) 18(8):884–93. 10.1016/S1473-3099(18)30397-9 29929783

[23] Corti D Misasi J Mulangu S Stanley DA Kanekiyo M Wollen S. Protective Monotherapy Against Lethal Ebola Virus Infection by a Potently Neutralizing Antibody. Science (80-) (2016) 351(6279):1339–42. 10.1126/science.aad5224 26917593

[24] Baum A Ajithdoss D Copin R Zhou A Lanza K Negron N. REGN-COV2 Antibodies Prevent and Treat SARS-CoV-2 Infection in Rhesus Macaques and Hamsters. Science (2020) 2402: (October):1–12. 10.1126/science.abe2402 PMC7857396 33037066

[25] Schaefer W Regula JT Bähner M Schanzer J Croasdale R Dürr H. Immunoglobulin Domain Crossover as a Generic Approach for the Production of Bispeci Fi C IgG Antibodies. Med Sci (2011) 108: (27):11187–92. 10.1073/pnas.109002108 PMC3131342 21690412

[26] Schlake T Thran M Fiedler K Heidenreich R Petsch B. mRNA : A Novel Avenue to Antibody Therapy? Mol Ther [Internet] (2019) 27(4):26–33. 10.1016/j.ymthe.2019.03.002 PMC6453519 30885573

[27] Oswald WB Geisbert TW Davis KJ Geisbert JB Sullivan NJ Jahrling PB. Neutralizing Antibody Fails to Impact the Course of Ebola Virus Infection in Monkeys. PLoS Pathog (2007) 3(1):0062–6. 10.1371/journal.ppat.0030009 PMC1779296 17238286

[28] Mbala-Kingebeni P Aziza A Di Paola N Wiley MR Makiala-Mandanda S Caviness K. Medical Countermeasures During the 2018 Ebola Virus Disease Outbreak in the North Kivu and Ituri Provinces of the Democratic Republic of the Congo: A Rapid Genomic Assessment. Lancet Infect Dis [Internet] (2019) 19(6):648–57. 10.1016/S1473-3099(19)30118-5 31000464

[29] Wang J Bardelli M Espinosa DA Pedotti M Ng T-S Bianchi S. A Human Bi-Specific Antibody Against Zika Virus With High Therapeutic Potential. Cell (2017) 171(1):229–41. Elsevier Enhanced Reader. 10.1016/j.cell.2017.09.002 PMC5673489 28938115

[30] De Gasparo R Pedotti M Simonelli L Nickl P Muecksch F Percivalle E. Bispecific Antibody Neutralizes Circulating SARS-CoV-2 Variants, Prevents Escape and Protects Mice From Disease 2 3. bioRxiv [Internet] (2021). 10.1101/2021.01.22.427567

[31] Klein C Schaefer W Regula JT Dumontet C Brinkmann U Bacac M. Engineering Therapeutic Bispecific Antibodies Using CrossMab Technology. Methods [Internet] (2019) 154:21–31. 10.1016/j.ymeth.2018.11.008 30453028

[32] Frei JC Nyakatura EK Zak SE Bakken RR Chandran K Dye JM. Bispecific Antibody Affords Complete Post-Exposure Protection of Mice From Both Ebola (Zaire) and Sudan Viruses. Sci Rep (2016) 6(January):2–11. 10.1038/srep19193 26758505 PMC4725817

[33] Wec AZ Nyakatura EK Herbert AS Howel KA Holtsberg FW Bakken RR. A “Trojan Horse” Bispecific-Antibody Strategy for Broad Protection Against Ebolaviruses. Science (80-) (2016) 354(6310):350–4. 10.1126/science.aag3267 PMC5647781 27608667

[34] Duehr J Wohlbold TJ Oestereich L Chromikova V Amanat F Rajendran M. Novel Cross-Reactive Monoclonal Antibodies Against Ebolavirus Glycoproteins Show Protection in a Murine Challenge Model. J Virol (2017) 91: (16):1–12. 10.1128/JVI.00652-17 PMC5533894 28592526

[35] Ovacik M Lin K. Tutorial on Monoclonal Antibody Pharmacokinetics and Its Considerations in Early Development. Clin Transl Sci (2018) 11(6):540–52. 10.1111/cts.12567 PMC6226118 29877608

[36] Liu Q Fan C Li Q Zhou S Huang W Wang L. Antibody-Dependent-Cellular-Cytotoxicity-Inducing Antibodies Significantly Affect the Post-Exposure Treatment of Ebola Virus Infection. Sci Rep (2017) 7(March):1–11. 10.1038/srep45552 28358050 PMC5372081

[37] Shields RL Namenuk AK Hong K Meng YG Rae J Briggs J. High Resolution Mapping of the Binding Site on Human IgG1 for Fcγri, Fcγrii, Fcγriii, and FcRn and Design of IgG1 Variants With Improved Binding to the Fcγr. J Biol Chem (2001) 276(9):6591–604. 10.1074/jbc.M009483200 11096108

[38] Saunders KO. Conceptual Approaches to Modulating Antibody Effector Functions and Circulation Half-Life. Front Immunol (2019) 10(JUN):1–20. 10.3389/fimmu.2019.01296 31231397 PMC6568213

[39] Pinto D Park YJ Beltramello M Walls AC Tortorici MA Bianchi S. Cross-Neutralization of SARS-CoV-2 by a Human Monoclonal SARS-CoV Antibody. Nat [Internet] (2020) 583(7815):290–5. 10.1038/s41586-020-2349-y 32422645

[40] Falzarano D Geisbert TW Feldmann H. Progress in Filovirus Vaccine Development: Evaluating the Potential for Clinical Use. Expert Rev Vaccines (2011) 10(1):63–77. 10.1586/erv.10.152 21162622 PMC3398800

[41] Bornholdt ZA Herbert AS Mire CE He S Robert W Wec AZ. A Two-Antibody Pan-Ebolavirus Cocktail Confers Broad Therapeutic Protection in Ferrets and Nonhuman Primates. Cell Host Microbe (2020) 25: (62):49–58. 10.1016/j.chom.2018.12.005 PMC6341996 30629918

[42] Lee JE Fusco ML Hessell AJ Oswald WB Burton DR Saphire EO. Structure of the Ebola Virus Glycoprotein Bound to an Antibody From a Human Survivor | Stanford Synchrotron Radiation Lightsource. Nat [Internet] (2008) 454(7201):177–82. 10.1038/nature07082 PMC2700032 18615077

[43] Volchkov VE Feldmann H Volchkova VA Klenk HD. Processing of the Ebola Virus Glycoprotein by the Proprotein Convertase Furin. Proc Natl Acad Sci U S A (1998) 95(10):5762–7. 10.1073/pnas.95.10.5762 PMC20453 9576958

[44] Sanchez A Trappier SG Mahy BWJ Peters CJ Nichol ST. The Virion Glycoproteins of Ebola Viruses are Encoded in Two Reading Frames and are Expressed Through Transcriptional Editing. Proc Natl Acad Sci U S A (1996) 93(8):3602–7. 10.1073/pnas.93.8.3602 PMC39657 8622982

[45] Volchkov VE Becker S Volchkova VA Ternovoj VA Kotov AN Netesov SV. GP mRNA of Ebola Virus Is Edited by the Ebola Virus Polymerase and by T7 and Vaccinia Virus Polymerases1. Virology (1995) 214(2):421–30. 10.1006/viro.1995.0052 8553543

[46] Wahl-Jensen VM Afanasieva TA Seebach J Ströher U Feldmann H Schnittler H-J. Effects of Ebola Virus Glycoproteins on Endothelial Cell Activation and Barrier Function. J Virol (2005) 79(16):10442–50. 10.1128/JVI.79.16.10442-10450.2005 PMC1182673 16051836

[47] Barrientos LG Martin AM Rollin PE Sanchez A. Disulfide Bond Assignment of the Ebola Virus Secreted Glycoprotein SGP. Biochem Biophys Res Commun (2004) 323(2):696–702. 10.1016/j.bbrc.2004.08.148 15369806

[48] Volchkova VA Dolnik O Martinez MJ Reynard O Volchkov VE. Genomic RNA Editing and its Impact on Ebola Virus Adaptation During Serial Passages in Cell Culture and Infection of Guinea Pigs. J Infect Dis (2011) 204(SUPPL. 3):941–6. 10.1093/infdis/jir321 21987773

[49] De La Vega MA Wong G Kobinger GP Qiu X. The Multiple Roles of sGP in Ebola Pathogenesis. Viral Immunol (2015) 28(1):3–9. 10.1089/vim.2014.0068 25354393 PMC4287119

[50] Maruyama T Parren PWHI Sanchez A Rensink I Rodriguez LL Khan AS. Recombinant Human Monoclonal Antibodies Against Ebola Virus. JID (1999) 179(1):235–9. 10.1086/514280 9988189

[51] Maruyama T Rodriguez LL Jahrling PB Sanchez A Khan AS Nichol ST. Ebola Virus Can Be Effectively Neutralized by Antibody Produced in Natural Human Infection. J Virol (1999) 73(7):6024–30. 10.1128/JVI.73.7.6024-6030.1999 PMC112663 10364354

[52] Ito H Watanabe S Takada A Kawaoka Y. Ebola Virus Glycoprotein: Proteolytic Processing, Acylation, Cell Tropism, and Detection of Neutralizing Antibodies. J Virol (2001) 75(3):1576–80. 10.1128/JVI.75.3.1576-1580.2001 PMC114066 11152533

[53] Takada A Watanabe S Okazaki K Kida H Kawaoka Y. Infectivity-Enhancing Antibodies to Ebola Virus Glycoprotein. J Virol (2001) 75(5):2324–30. 10.1128/JVI.75.5.2324-2330.2001 PMC114815 11160735

[54] Shahhosseini S Das D Qiu X Feldmann H Jones SM Suresh MR. Production and Characterization of Monoclonal Antibodies Against Different Epitopes of Ebola Virus Antigens. J Virol Methods (2007) 143(1):29–37. 10.1016/j.jviromet.2007.02.004 17368819

[55] Dowling W Thompson E Badger C Mellquist JL Garrison AR Smith JM. Influences of Glycosylation on Antigenicity, Immunogenicity, and Protective Efficacy of Ebola Virus GP DNA Vaccines. J Virol (2007) 81(4):1821–37. 10.1128/JVI.02098-06 PMC1797596 17151111

[56] Martinez O Tantral L Mulherkar N Chandran K Basler CF. Impact of Ebola Mucin-Like Domain on Antiglycoprotein Antibody Responses Induced by Ebola Virus-Like Particles. J Infect Dis (2011) 204(SUPPL. 3):825–32. 10.1093/infdis/jir295 PMC3189980 21987758

[57] Olal D Kuehne AI Bale S Halfmann P Hashiguchi T Fusco ML. Structure of an Antibody in Complex With Its Mucin Domain Linear Epitope That Is Protective Against Ebola Virus. J Virol (2012) 86(5):2809–16. 10.1128/JVI.05549-11 PMC3302272 22171276

[58] Qiu X Alimonti JB Melito PL Fernando L Ströher U Jones SM. Characterization of Zaire Ebolavirus Glycoprotein-Specific Monoclonal Antibodies. Clin Immunol [Internet] (2011) 141(2):218–27. 10.1016/j.clim.2011.08.008 21925951

[59] Wilson JA Hevey M Bakken R Guest S Bray M Schmaljohn AL. Epitopes Involved in Antibody-Mediated Protection From Ebola Virus. Science (80-) (2000) 287(5458):1664–6. 10.1126/science.287.5458.1664 10698744

[60] Francica JR Varela-Rohena A Medvec A Plesa G Riley JL Bates P. Steric Shielding of Surface Epitopes and Impaired Immune Recognition Induced by the Ebola Virus Glycoprotein. PLoS Pathog (2010) 6(9):1–14. 10.1371/journal.ppat.1001098 PMC2936550 20844579

[61] Zampieri CA Sullivan NJ Nabel GJ. Immunopathology of Highly Virulent Pathogens: Insights From Ebola Virus. Nat Immunol (2007) 8(11):1159–64. 10.1038/ni1519 PMC7097212 17952040

[62] Rimoin AW Lu K Bramble MS Steffen I Doshi RH Hoff NA. Ebola Virus Neutralizing Antibodies Detectable in Survivors of Theyambuku, Zaire Outbreak 40 Years After Infection. J Infect Dis (2018) 217(2):223–31. 10.1093/infdis/jix584 PMC5853670 29253164

[63] Rowe AK Bertolli J Khan AS Mukunu R Muyembe-Tamfum JJ Bressler D. Clinical, Virologic, and Immunologic Follow-Up of Convalescent Ebola Hemorrhagic Fever Patients and Their Household Contacts, Kikwit, Democratic Republic of the Congo. J Infect Dis (1999) 179(SUPPL. 1):28–35. 10.1086/514318 9988162

[64] Murin CD Wilson IA Ward AB. Antibody Responses to Viral Infections: A Structural Perspective Across Three Different Enveloped Viruses. Nat Microbiol (2019) 4: (5):734–47. 10.1038/s41564-019-0392-y PMC6818971 30886356

[65] FDA. First FDA-Approved Vaccine for the Prevention of Ebola Virus Disease, Marking a Critical Milestone in Public Health Preparedness and Response (2021). Available at: https://www.fda.gov/news-events/press-announcements/first-fda-approved-vaccine-prevention-ebola-virus-disease-marking-critical-milestone-public-health.

[66] Henao-Restrepo AM Camacho A Longini IM Watson CH Edmunds WJ Egger M. Efficacy and Effectiveness of an rVSV-Vectored Vaccine in Preventing Ebola Virus Disease: Final Results From the Guinea Ring Vaccination, Open-Label, Cluster-Randomised Trial (Ebola Ça Suffit)! Lancet (2017) 389(10068):505–18. 10.1016/S0140-6736(16)32621-6 PMC5364328 28017403

[67] WHO. Preliminary Results on the Efficacy of rVSV-ZEBOV-GP Ebola Vaccine Using the Ring Vaccination Strategy in the Control of an Ebola Outbreak in the Democratic Republic of the Congo: An Example of Integration of Research Into Epidemic Response. Available at https://www.who.intebola-ring-vaccination-results-12-april-2019.pdf (who.int)

[68] Wiedemann A Foucat E Hocini H Lefebvre C Hejblum BP Durand M. Long-Lasting Severe Immune Dysfunction in Ebola Virus Disease Survivors. Nat Commun [Internet] (2020) 11(1):1–11. 10.1038/s41467-020-17489-7 PMC7381622 32709840

[69] Luke T Bennett RS Gerhardt DM Burdette T Postnikova E Mazur S. Fully Human Immunoglobulin G From Transchromosomic Bovines Treats Nonhuman Primates Infected With Ebola Virus Makona Isolate. J Infect Dis (2018) 218(Suppl 5):S636–48. 10.1093/infdis/jiy377 PMC6249570 30010950

[70] Cross RW Bornholdt ZA Prasad AN Geisbert JB Borisevich V Agans KN. Prior Vaccination With rVSV-ZEBOV Does Not Interfere With But Improves Efficacy of Postexposure Antibody Treatment. Nat Commun [Internet] (2020) 11(1):1–8. 10.1038/s41467-020-17446-4 PMC7385100 32719371

[71] Fausther-Bovendo H Mulangu S Sullivan NJ. Ebolavirus Vaccines for Humans and Apes. Curr Opin Virol [Internet] (2012) 2(3):324–9. 10.1016/j.coviro.2012.04.003 PMC3397659 22560007

[72] Graham BS Sullivan NJ. Emerging Viral Diseases From a Vaccinology Perspective: Preparing for the Next Pandemic Review-Article. Nat Immunol [Internet] (2018) 19(1):20–8. 10.1038/s41590-017-0007-9 PMC7097586 29199281

[73] Zhu N Zhang D Wang W Li X Yang B Song J. A Novel Coronavirus From Patients With Pneumonia in China, 2019. N Engl J Med (2020) 382(8):727–33. 10.1056/NEJMoa2001017 PMC7092803 31978945

[74] Markham A. REGN-EB3: First Approval. Drugs [Internet] (2021) 81(1):175–8. 10.1007/s40265-020-01452-3 PMC7799152 33432551

[75] Gilchuk P Kuzmina N Ilinykh PA Huang K Gunn BM Bryan A. Multifunctional Pan-Ebolavirus Antibody Recognizes a Site of Broad Vulnerability on the Ebolavirus Glycoprotein. Immun [Internet] (2018) 49(2):363–374.e10. 10.1016/j.immuni.2018.06.018 PMC6104738 30029854

[76] Holtsberg FW Shulenin S Vu H Howell KA Patel SJ Gunn B. Pan-Ebolavirus and Pan-Filovirus Mouse Monoclonal Antibodies: Protection Against Ebola and Sudan Viruses. J Virol (2016) 90(1):266–78. 10.1128/JVI.02171-15 PMC4702560 26468533

